# Diazepam Accelerates GABA_A_R Synaptic Exchange and Alters Intracellular Trafficking

**DOI:** 10.3389/fncel.2019.00163

**Published:** 2019-04-26

**Authors:** Joshua M. Lorenz-Guertin, Matthew J. Bambino, Sabyasachi Das, Susan T. Weintraub, Tija C. Jacob

**Affiliations:** ^1^Department of Pharmacology and Chemical Biology, School of Medicine, University of Pittsburgh, Pittsburgh, PA, United States; ^2^Department of Biochemistry and Structural Biology, University of Texas Health Science Center at San Antonio, San Antonio, TX, United States

**Keywords:** GABA_A_R, benzodiazepine, trafficking, gephyrin, mass spectrometry, diazepam, inhibitory synapse

## Abstract

Despite 50+ years of clinical use as anxiolytics, anti-convulsants, and sedative/hypnotic agents, the mechanisms underlying benzodiazepine (BZD) tolerance are poorly understood. BZDs potentiate the actions of gamma-aminobutyric acid (GABA), the primary inhibitory neurotransmitter in the adult brain, through positive allosteric modulation of γ2 subunit containing GABA type A receptors (GABA_A_Rs). Here we define key molecular events impacting γ2 GABA_A_R and the inhibitory synapse gephyrin scaffold following initial sustained BZD exposure *in vitro* and *in vivo*. Using immunofluorescence and biochemical experiments, we found that cultured cortical neurons treated with the classical BZD, diazepam (DZP), presented no substantial change in surface or synaptic levels of γ2-GABA_A_Rs. In contrast, both γ2 and the postsynaptic scaffolding protein gephyrin showed diminished total protein levels following a single DZP treatment *in vitro* and in mouse cortical tissue. We further identified DZP treatment enhanced phosphorylation of gephyrin Ser270 and increased generation of gephyrin cleavage products. Selective immunoprecipitation of γ2 from cultured neurons revealed enhanced ubiquitination of this subunit following DZP exposure. To assess novel trafficking responses induced by DZP, we employed a γ2 subunit containing an N terminal fluorogen-activating peptide (FAP) and pH-sensitive green fluorescent protein (γ2^pH^FAP). Live-imaging experiments using γ2^pH^FAP GABA_A_R expressing neurons identified enhanced lysosomal targeting of surface GABA_A_Rs and increased overall accumulation in vesicular compartments in response to DZP. Using fluorescence resonance energy transfer (FRET) measurements between α2 and γ2 subunits within a GABA_A_R in neurons, we identified reductions in synaptic clusters of this subpopulation of surface BZD sensitive receptor. Additional time-series experiments revealed the gephyrin regulating kinase ERK was inactivated by DZP at multiple time points. Moreover, we found DZP simultaneously enhanced synaptic exchange of both γ2-GABA_A_Rs and gephyrin using fluorescence recovery after photobleaching (FRAP) techniques. Finally we provide the first proteomic analysis of the BZD sensitive GABA_A_R interactome in DZP vs. vehicle treated mice. Collectively, our results indicate DZP exposure elicits down-regulation of gephyrin scaffolding and BZD sensitive GABA_A_R synaptic availability via multiple dynamic trafficking processes.

## Introduction

GABA_A_Rs are ligand-gated ionotropic chloride (Cl^-^) channels responsible for the majority of fast inhibitory neurotransmission in the adult CNS. The most prevalent synaptic GABA_A_R subtype is composed of two α, two β, and a γ2 subunit forming a heteropentamer (Uusi-Oukari and Korpi, 2010). Benzodiazepines (BZDs) are a widely used clinical sedative-hypnotic drug class that selectively bind between the interface of a GABA_A_R γ2 subunit and either an α1/2/3/5 subunit (Vinkers and Olivier, 2012). Receptors containing these α subunits are considered to be primarily synaptic, with the exception of α5, which is localized both synaptically and extrasynaptically (Brady and Jacob, 2015). Positive allosteric modulation by BZD enhances GABA_A_R inhibition by increasing the binding affinity of GABA and increasing channel opening frequency (Uusi-Oukari and Korpi, 2010). Recent cryo-electron microscopy publications have provided unprecedented structural and pharmacological information about benzodiazepine-sensitive GABA_A_Rs and the benzodiazepine binding site (Phulera et al., 2018; Zhu et al., 2018; Laverty et al., 2019; Masiulis et al., 2019) reinvigorating interest in the molecular actions of BZD drugs. It is known that the potentiating effect of BZDs are lost after prolonged or high dose acute exposure (Tietz et al., 1989; Holt et al., 1999), characterized first by a loss of sedative/hypnotic activity followed by the anti-convulsant properties behaviorally (Lister and Nutt, 1986; Wong et al., 1986; File et al., 1988; Bateson, 2002). The induction of BZD tolerance occurs in part due to the uncoupling of allosteric actions between GABA and BZD (Gallager et al., 1984; Marley and Gallager, 1989), a process that appears to rely on GABA_A_R receptor internalization (Ali and Olsen, 2001; Gutierrez et al., 2014). We have previously shown that 24 h BZD treatment leads to decreased surface and total levels of the α2 subunit in cultured hippocampal neurons that was dependent on lysosomal-mediated degradation (Jacob et al., 2012); however, the process by which the α2 subunit is selectively targeted to lysosomes is still unknown. GABA_A_R subunit ubiquitination and subsequent degradation at proteasomes or lysosomes modulates cell surface expression of receptors (Saliba et al., 2007; Arancibia-Carcamo et al., 2009; Crider et al., 2014; Jin et al., 2014; Di et al., 2016). Ubiquitination of the γ2 subunit is the only currently known mechanism identified to target internalized surface GABA_A_Rs to lysosomes (Arancibia-Carcamo et al., 2009).

Another major regulator of GABA_A_R efficacy is postsynaptic scaffolding. Confinement at synaptic sites maintains receptors at GABA axonal release sites for activation. Furthermore, this limits receptor diffusion into the extrasynaptic space where internalization occurs (Bogdanov et al., 2006; Gu et al., 2016). The scaffolding protein gephyrin is the main organizer of GABA_A_R synaptic localization and density, as gephyrin knock-down and knock-out models show dramatic reductions in γ2- and α2-GABA_A_R clustering (Kneussel et al., 1999; Jacob et al., 2005). Evidence suggests gephyrin interacts directly with GABA_A_R α1, α2, α3, α5, β2, and β3 subunits (Tretter et al., 2008; Maric et al., 2011; Mukherjee et al., 2011; Kowalczyk et al., 2013; Tyagarajan and Fritschy, 2014; Brady and Jacob, 2015). Gephyrin recruitment is involved in inhibitory long term potentiation (Petrini et al., 2014; Flores et al., 2015), while its dispersal coincides with GABA_A_R diffusion away from synapses (Jacob et al., 2005; Bannai et al., 2009). Extensive post-translational modifications influence gephyrin function (Zacchi et al., 2014; Ghosh et al., 2016). Accordingly, expression of gephyrin phosphorylation site mutants revealed complex effects on GABA_A_R diffusion and gephyrin ultrastructure and scaffolding (Ghosh et al., 2016; Battaglia et al., 2018). Phosphorylation at the gephyrin serine 270 (Ser270) site has been particularly characterized to negatively modulate scaffold clustering and density, in part by enhancing calpain-1 protease mediated degradation of gephyrin (Tyagarajan et al., 2013). Given the well-established interdependent relationship between gephyrin and the γ2 subunit in maintaining receptor synaptic integrity (Essrich et al., 1998; Kneussel et al., 1999; Schweizer et al., 2003; Alldred et al., 2005; Jacob et al., 2005; Li et al., 2005), impaired postsynaptic scaffolding should affect both pre-existing and newly inserted GABA_A_R clustering and ultimately the efficacy of inhibitory neurotransmission. Thus a central unanswered question is if BZD exposure causes changes in gephyrin phosphorylation or protein levels.

Here we demonstrate that 12–24 h treatment with the BZD diazepam (DZP) leads to a reduction in total γ2 subunit and full-length gephyrin levels *in vitro* and *in vivo*. This reduction occurred coincident with enhanced γ2 subunit ubiquitination, but resulted in no significant change in overall γ2 surface levels. Using our recently published dual fluorescent BZD-sensitive GABA_A_R reporter (γ2^pH^FAP), we further show that cell surface γ2-GABA_A_Rs are more frequently targeted to lysosomes after DZP exposure. Forester resonance energy transfer (FRET) experiments further confirmed specific loss of synaptic α2/γ2 GABA_A_R levels following DZP treatment. The scaffolding protein gephyrin also demonstrated augmented phosphorylation at Ser270, increased cleavage and was significantly decreased in membrane and cytosolic compartments. Fluorescence recovery after photobleaching (FRAP) assays identified that DZP treatment increased the simultaneous recovery of γ2-GABA_A_R and gephyrin at synaptic sites, indicating reduced receptor confinement and accelerated exchange between the synaptic and extrasynaptic GABA_A_R pool. This process was reversed by the BZD site antagonist Ro 15-1788. Lastly, co-immunoprecipitation, quantitative mass spectrometry and bioinformatics analysis revealed shifts in the γ2-GABA_A_R interactome toward trafficking pathways *in vivo*. Together, these data suggest that DZP exposure causes a compensatory decrease in inhibitory neurotransmission by reducing BZD-sensitive GABA_A_R and gephyrin confinement at synapses and via ubiquitination and lysosomal targeting of γ2.

## Materials and Methods

### Cell Culture, Transfection, Expression Constructs and Mice

Cortical neurons were prepared from embryonic day 18 rats and nucleofected with constructs at plating (Amaxa). The γ2^pH^FAP construct was characterized in Lorenz-Guertin et al. (2017) and RFP-gephyrin was described in Brady and Jacob (2015). The γ2^RFP^ construct was generated by PCR cloning and fully sequenced: the red fluorescent protein mCherry replaced pHluorin in the previously published γ2^pHGFP^ construct (Jacob et al., 2005). GFP-ubiquitin was a gift from Nico Dantuma (Addgene plasmid # 11928) (Dantuma et al., 2006). 8–10 weeks old male C57BL/6J mice (Jackson Laboratory) were maintained on a reverse 12 h dark/light schedule. Mouse cortical brain tissue was collected and flash frozen 12 h after I.P. injection with either vehicle or diazepam [in 40% PEG, 10% EtOH, 5% Na Benzoate, 1.5% Benzyl alcohol (Hospira)]. All procedures were approved by the University of Pittsburgh Institutional Animal Care and Use Committee.

### Reagents, Antibodies, and MG Dye

Diazepam (cell culture, Sigma; injections, Hospira); Ro 15-1788 (Tocris Bioscience); calpain-1 inhibitor MDL-28170 (Santa Cruz); L-glutamic acid (Tocris Bioscience). Primary antibodies: rabbit GAPDH (WB) (14C10, Cell Signaling); guinea pig GAD-65 (IF) (198104, Synaptic Systems); rabbit γ2 GABA_A_R subunit (IF, WB, and IP) (224003, Synaptic Systems); rabbit gephyrin (WB) (sc-14003, Santa Cruz); rabbit gephyrin (IF, total) (147002, Synaptic Systems) (antibody validation Supplementary Figure S1); mouse gephyrin mAb7a (IF, phospho) (147011, Synaptic Systems); chicken GFP (WB) (GFP-1020, Aves); rabbit (P)ERK (WB) (4370, Cell Signaling); mouse ERK (WB) (9107, Cell Signaling); rabbit (P)GSK3β (WB) (9322, Cell Signaling); rabbit GSK3β (WB) (9315. Cell Signaling); rabbit CDK5 (WB) (2506, Cell Signaling). MG-BTau dye prepared as in Lorenz-Guertin et al. (2017).

### Fixed and Live-Imaging

Measurements were made on days *in vitro* (DIV) 15–19 cortical neurons. Live-imaging performed in Hepes-buffered saline (HBS), containing the following (in mM): 135 NaCl, 4.7 KCl, 10 Hepes, 11 glucose, 1.2 MgCl_2_, and 2.5 CaCl_2_ (adjusted to pH 7.4 with NaOH). Images were acquired using a Nikon A1 confocal microscope with a 60× oil objective (N.A., 1.49) at 3× zoom. Data were analyzed in NIS Elements software (Nikon, N.Y.). Measurements were taken from whole cell or averaged from three dendritic 10 μm regions of interest (ROI) per cell. For fixed imaging, media was quickly removed and coverslips were washed twice with Dulbecco’s Phosphate Buffered Saline (DPBS) and immediately fixed with 4% paraformaldehyde and then blocked in PBS containing 10% fetal bovine serum and 0.5% bovine serum albumin. Surface antibody staining was performed under non-permeabilized conditions overnight at 4°C. Intracellular staining was performed overnight at 4°C following 0.2% Triton-X permeabilization for 10 min in blocking solution. Synaptic sites were determined during analysis by binary thresholds and colocalization with GAD-65. Extrasynaptic intensity was measured by taking the total dendrite ROI sum intensity minus background and synaptic fluorescence intensity. Dendritic fluorescence was measured using binary thresholds. Experimental conditions were blinded during image acquisition and analysis. The ROUT test (*Q* = 1%) or Grubbs’ Test (alpha = 0.05) was used to remove a single outlier from a data set.

### Lysosomal Targeting Assay

Neuron surface and lysosomal-association assays utilized MG-BTau dye for surface receptor pulse-labeling. DIV 15–16 neurons were treated with vehicle or DZP for 8–12 h, then pulse labeled with 100 nM MG-BTau for 2 min at room temperature in HBS. Neurons were then washed 5× times with HBS and returned to conditioned media ± DZP for 1 h. To identify lysosomal targeting, 50 nM LysoTracker Blue DND-22 (Life Technologies) and the lysosomal inhibitor, Leupeptin (200 μM Amresco), was added 30 min prior to imaging. Following incubation, neurons were washed and imaged in 4°C HBS. Two–three neurons were immediately imaged per culture dish within 10 min of washing. For image analysis, independent ROIs were drawn to capture the soma, three 10 μm sections of dendrite and the whole cell. Binary thresholds and colocalization measurements were performed to identify MG-BTau, pHGFP synaptic GABA_A_R clusters and lysosomes. Total surface pHGFP expression was determined by taking the entire cell surface signal following background subtraction.

### NH_4_Cl Intracellular Imaging

DIV 15–16 neurons were washed and continuously perfused with HBS + treatment at room temperature. Multiposition acquisition was used to image 2–3 neurons per dish. An initial image was taken to identify surface γ2^pH^FAP GABA_A_Rs. Neurons were then perfused with NH_4_Cl solution to collapse the cellular pH gradient and were reimaged. NH_4_Cl solution (in mM): 50 NH_4_Cl, 85 NaCl, 4.7 KCl, 10 Hepes, 11 glucose, 1.2 MgCl_2_, and 2.5 CaCl_2_ (adjusted to pH 7.4 with NaOH). pHGFP intensity was measured following background subtraction and smoothing. Surface/total levels were determined by dividing the first image (surface only) from the second image (total). The spot detection tool in Nikon Elements was used to selectively count larger intracellular vesicles positive for γ2^pH^FAP. A stringent threshold was set to identify brightly fluorescent circular objects with a circumference of approximately 0.75 μm. Values reflect new vesicle objects that were only seen after NH_4_Cl perfusion (second image – first image).

### Intermolecular FRET Imaging, Characterization and Analysis

The α2 pHGFP (α2^pH^) construct was previously published (Tretter et al., 2008) and the γ2^RFP^ construct was generated by PCR cloning and fully sequenced. DIV 15–16 neurons were treated with Veh or DZP for 20–28 h, then washed and continuously perfused with HBS at room temperature. Images were acquired with a 60× objective at 2× zoom. For each cell, an initial image was acquired containing two channels to identify surface α2^pH^ (excited by 488 laser, emission band pass filter 500–550) and γ2^RFP^ participating in FRET (excited 488 FRET, emission band pass filter 575–625 nm, FRET channel). A second, single channel image was taken immediately following with only 561 nm excitation to reveal total γ2^RFP^ levels (excited by 561 laser, emission band pass filter 575–625 nm). For synaptic quantifications, binary thresholding based on intensity was applied with smoothing and size exclusion (0–3 μm) factors. FRET and 561 channel binaries shared identical minimum and maximum binary threshold ranges. Individual synaptic ROIs were created to precisely target and measure synaptic clusters containing both α2^pH^ and γ2^RFP^. Manual trimming and single pixel removal were used to remove signal not meeting the criteria of a receptor cluster. Restriction criteria were applied in the following order: (1) at least 15 synapses measured per cell, (2) FRET γ2^RFP^: raw γ2^RFP^ sum intensity ratio must be less than one, (3) synaptic α2^pH^ mean intensity of at least 500, and (4) α2^pH^ sum intensity limit of 300% of average sum intensity. ROI data was then normalized to vehicle control as percent change. The percentage of RFP participating in FRET was also calculated using FRET RFP:Total RFP ratio.

Fluorescence resonance energy transfer activity was directly assessed by acceptor (γ2^RFP^) photobleaching. Photobleaching ROIs were implemented on 2 synapses per cell. Pre-bleaching images were acquired every 5 s, followed by a γ2^RFP^ photobleaching event using 80% 561 nm laser power. After photobleaching, image capturing resumed without delay using pre-bleach laser power settings for 2 min. Image analysis incorporated background subtraction and the measurement of percent change in α2^pH^/FRET γ2^RFP^ ratio over the time course. FRET efficacy measurements compared directly adjacent α2^pH^ and γ2^RFP^ subunits in a GABA_A_R complex. Live-imaging with perfusion of pH 6.0 extracellular imaging saline solution (MES) was used to quench the pH-dependent GFP fluorescence from the α2^pH^ donor fluorophores and show the dependence of FRET on surface α2^pH^ fluorescence. Acidic extracellular saline solution, MES solution pH 6.0 (in mM): 10 MES, 135 NaCl, 4.7 KCl, 11 glucose, 1.2 MgCl_2_, and 2.5 CaCl_2_ (adjusted to pH 7.4 with NaOH). Images were collected under HBS conditions for 1 min at 20 s intervals, and then followed by a 2 min MES wash with the same imaging interval to quench donor emissions. FRET RFP mean intensity was measured under both conditions and normalized to HBS. Percent or fold change in FRET RFP emissions were reported as indicated.

### Synaptic Exchange Rate FRAP Imaging

Neurons were washed and media was replaced with HBS + treatment. Imaging was performed in an enclosed chamber at 37°C. An initial image was taken for baseline standardization. Photobleaching was performed by creating a stimulation ROI box encompassing two or more dendrites. This stimulation region was photobleached using the 488 and 561 lasers at 25% power for 1 min. The same stimulation ROI was used for every cell in an experiment. Immediately following photobleaching, 10 nM MG-Tau dye was added to the cell culture dish to re-identify surface synaptic GABA_A_R clusters. Time-lapse imaging was then started every 2 min for 60 min. During image analysis, objects were only considered synaptic if they demonstrated colocalization with γ2^pH^FAP pHGFP signal, RFP-gephyrin signal and had obvious surface MG-BTau fluorescence. ROIs were drawn measuring the rate of fluorescence recovery at 4–8 synaptic sites and one extrasynaptic site (10 μm long region; Bezier tool) per cell. For data analysis, synapse post-bleach fluorescence intensity time point data was first normalized to pre-bleach fluorescence intensity (post-bleach/pre-bleach). Normalized synapse post-bleach data was then calculated as percent change from t0 [(tx/t0)^∗^100, where *x* = min]. Individual synapses were then averaged to calculate fluorescence recovery and statistically significant changes across time points.

### Western Blot and Immunoprecipitation

Protein concentration was determined by BCA protein assay for all biochemistry. Neurons were lysed in denaturing buffer for immunoprecipitation: 50 mM Tris HCl, 1 mM EDTA, 1% SDS, 2 mM Na3VO4, 10 mM NaF, 50 mM *N*-ethylmaleimide, protease inhibitor cocktail (Sigma). Lysates were sonicated and heated at 50°C for 20 min, then diluted 1:5 in RIPA buffer (50 mM Tris HCl pH 7.6, 150 mM NaCl, 1% Igepal, 0.5% Sodium deoxycholate, 1 mM EDTA, 2 mM Na3VO4, 10 mM NaF, 50 mM *N*-ethylmaleimide, protease inhibitor cocktail). Standard immunoprecipitation procedures were performed using overnight incubation with γ2 subunit antibody or rabbit IgG (sci2027; Sigma), 1 h incubation with Protein A Sepharose 4B beads (Invitrogen), three RIPA buffer washes, and loading for SDS-PAGE. After electrophoresis and transfer to nitrocellulose membrane, samples were probed with primary antibody overnight followed by the appropriate horseradish peroxide (HRP)-coupled secondary antibody.

### Membrane and Subcellular Fractionation

Cultured neurons were lysed using fractionation buffer: 50 mM Tris-HCl, 50 mM NaCl, 1 mM EDTA, 2 mM Na3VO4, 10 mM NaF, 320 mM sucrose, 0.25% igepal, and protease inhibitor cocktail. Lysates were spun at 88,881 *g* for 30 min at 4°C to separate pellet (membrane) from supernatant (cytosol). Fraction integrity was tested by localization specific markers in all experiments (Supplementary Figure S2 and data not shown).

### Co-immunoprecipitation

Mice were intraperitoneally (I.P.) injected with vehicle control or 10 mg/kg DZP and sacrificed 12 h post-injection (*n* = 4 mice per treatment). Mouse cortical tissue was homogenized in co-IP buffer (50 mM Tris HCl pH 7.6, 50 mM NaCl, 0.25% Igepal, 1 mM EDTA, 2 mM Na3VO4, 10 mM NaF, 50 mM *N*-ethylmaleimide, and Sigma protease inhibitor cocktail) using a Dounce homogenizer. Tissue was solubilized with end-over-end mixing at 4°C for 15 min, and then spun at 1,000 g to remove non-solubilized fractions. Each immunoprecipitation tube contained 375 μg of tissue lysate brought up to 1 ml volume using co-IP buffer. Lysates were precleared using Protein A Sepharose 4B beads (Invitrogen) for 1 h at 4°C. Lysate was then immunoprecipitated overnight with 2.5 μg rabbit γ2 subunit antibody (224003, Synaptic Systems) or 2.5 μg rabbit IgG (2027, Santa Cruz). The next day, 40 μl Protein A Sepharose slurry was added and mixed for 2 h at 4°C on a nutator. Beads were then washed 3× at 4°C on a nutator in 1 ml co-IP buffer. Beads were denatured with SDS-PAGE loading buffer [Laemmli Sample buffer (Bio-Rad) + β-mercaptoethanol] with heat at 70°C for 10 min and intermittent vortexing. Two immunoprecipitation reactions were performed per animal and were pooled into a single tube without beads to be used for downstream in-gel digestion.

### Mass Spectrometry and Data Processing

Immunoprecipitated proteins were separated by electrophoresis in Criterion XT MOPS 12% SDS-PAGE reducing gels (Bio-Rad), with subsequent protein visualization by staining with Coomassie blue. Each gel lane was divided into six slices. After de-staining, proteins in the gel slices were reduced with TCEP [tris(2-carboxyethyl)phosphine hydrochloride] and then alkylated with iodoacetamide before digestion with trypsin (Promega). HPLC-electrospray ionization-tandem mass spectrometry (HPLC-ESI-MS/MS) was accomplished by data-dependent acquisition on a Thermo Fisher Orbitrap Fusion Lumos Tribrid mass spectrometer. Mascot (Matrix Science; London, United Kingdom) was used to search the MS files against the mouse subset of the UniProt database combined with a database of common contaminants. Subset searching of the Mascot data, determination of probabilities of peptide assignments and protein identifications, were accomplished by Scaffold (v 4.8.4, Proteome Software). MS data files for each entire gel lane were combined via the “MudPIT” option. Identification criteria were: minimum of two peptides; 96% peptide threshold; 1% FDR; 99% protein threshold. One vehicle- and one DZP-treated animal were removed from analysis due to insufficient γ2 subunit pulldown relative to all other groups. *N* = 3 animals per condition were used for downstream analysis. Protein clustering was applied in Scaffold and weighted spectrum values and exclusive unique peptides were exported for manual excel analysis. Student’s *t*-test analysis was performed using relative fold change (ratio) of DZP compared to vehicle group. In some cases peptides were only detected in vehicle or DZP treated groups, resulting in DZP/V ratio values of zero or undefined error (cannot divide by zero). These were annotated as NF-DZP (not found in DZP samples) or NF-V (not found in vehicle samples) in the tables.

### Bioinformatics Analysis

Ingenuity Pathways Analysis (IPA) (Ingenuity Systems) was used for cellular pathway analysis. Relative fold levels of DZP proteins compared to vehicle were used for analysis. To be suitable for IPA analysis, proteins NF-DZP were assigned a value of -1E+99, while proteins NF-V were assigned a value of 1E+99. Significant enrichment in protein networks were calculated by right tailed Fisher’s exact test. *Z*-score analysis is a statistical measure of an expected relationship direction and observed protein/gene expression to predict pathway activation or inhibition. IPA core analysis was searched to determine direct and indirect relationships within 35 molecules per network and 25 networks per analysis. All data repositories available through IPA were used to determine experimentally observed and highly predicted interactions occurring in mammalian tissue and cell lines. Ratio data were converted to fold change values in IPA, where the negative inverse (-1/*x*) was taken for values between 0 and 1, while ratio values greater than 1 were not affected. Proteins found to be enhanced in their association with γ2 (Table 1) were searched in the Mus musculus GO Ontology database (released 2018-10-08) for GO biological process and GO molecular function and analyzed by the PANTHER overrepresentation test; significance was determined using Fisher’s Exact with Bonferroni correction for multiple testing.

**Table 1:** Proteins demonstrating increased association with γ2-GABA_A_Rs after DZP *in vivo* by mass spectrometry.

Ratio DZP/V	*P*-value	UniProtKB	Gene ID	Entrez gene name	Location	Type(s)
9.6	8.9E-02	Q14BI2	GRM2	Glutamate metabotropic receptor 2	Plasma Membrane	G-protein coupled receptor
9.5	4.3E-02	P12960	CNTN1	Contactin 1	Plasma Membrane	Enzyme
7.0	5.9E-02	P11276	FN1	Fibronectin 1	Extracellular Space	Enzyme
5.4	2.7E-02	E9Q4P0	KXD1	KxDL motif containing 1	Cytoplasm	Other
5.4	3.1E-02	Q62277	SYP	Synaptophysin	Cytoplasm	Transporter
5.0	5.0E-03	Q9QXY6	EHD3	EH domain containing 3	Cytoplasm	Other
5.0	7.8E-02	P48774	GSTM3	Glutathione *S*-transferase mu 3	Cytoplasm	Enzyme
4.9	1.9E-05	P38647	HSPA9	Heat shock protein family A (Hsp70) member 9	Cytoplasm	Other
4.7	6.4E-02	Q91V41	RAB14	RAB14, member RAS oncogene family	Cytoplasm	Enzyme
4.2	6.4E-02	P48758	CBR1	Carbonyl reductase 1	Cytoplasm	Enzyme
4.2	7.2E-02	Q8K3F6	KCNQ3	Potassium voltage-gated channel subfamily Q member 3	Plasma Membrane	Ion channel
4.2	3.3E-02	A0A0R4J036	Nefm	Neurofilament, medium polypeptide	Plasma Membrane	Other
4.0	7.9E-02	Q921I1	TF	Transferrin	Extracellular Space	Transporter
3.8	8.6E-02	Q9CYZ2	TPD52L2	Tumor protein D52 like 2	Cytoplasm	Other
3.3	4.2E-02	Q99KI0	ACO2	Aconitase 2	Cytoplasm	Enzyme
2.6	8.0E-02	Q9EQF6	DPYSL5	Dihydropyrimidinase like 5	Cytoplasm	Enzyme
2.4	2.2E-02	P56480	ATP5F1B	ATP synthase F1 subunit beta	Cytoplasm	Transporter
2.4	9.2E-02	P46096	SYT1	Synaptotagmin 1	Cytoplasm	Transporter
2.4	3.7E-02	Q6P1J1	CRMP1	Collapsin response mediator protein 1	Cytoplasm	Enzyme
2.2	3.8E-02	Q9DB20	ATP5PO	ATP synthase peripheral stalk subunit OSCP	Cytoplasm	Transporter
1.9	N.A.	P61027	RAB10	RAB10, member RAS oncogene family	Cytoplasm	Enzyme
1.8	9.6E-02	P63017	HSPA8	Heat shock protein family A (Hsp70) member 8	Cytoplasm	Enzyme
1.8	1.8E-02	P63011	RAB3A	RAB3A, member RAS oncogene family	Cytoplasm	Enzyme
1.8	1.3E-02	P17426-2	AP2A1	Adaptor related protein complex 2 subunit alpha 1	Cytoplasm	Transporter
1.7	9.1E-02	P18760	CFL1	Cofilin 1	Nucleus	Other
1.7	8.2E-02	Q9Z2I9	SUCLA2	Succinate-CoA ligase ADP-forming beta subunit	Cytoplasm	Enzyme
1.7	4.0E-02	P63328	PPP3CA	Protein phosphatase 3 catalytic subunit alpha	Cytoplasm	Phosphatase
1.7	7.1E-02	Q8R191	SYNGR3	Synaptogyrin 3	Plasma Membrane	Other
1.6	5.7E-02	Q8BHJ7	GABRA5	Gamma-aminobutyric acid type A receptor alpha5 subunit	Plasma Membrane	Ion channel
1.6	1.8E-02	O35129	PHB2	Prohibitin 2	Cytoplasm	Transcription regulator
1.6	1.6E-02	P61982	YWHAG	Tyrosine 3-monooxygenase/tryptophan 5-monooxygenase activation protein gamma (14-3-3 gamma)	Cytoplasm	Other
1.5	5.3E-02	P07901	HSP90AA1	Heat shock protein 90 alpha family class A member 1	Cytoplasm	Enzyme
1.5	7.8E-03	P67778	PHB	Prohibitin	Nucleus	Transcription regulator
1.5	8.1E-02	Q3UGC7	EIF3J	Eukaryotic translation initiation factor 3 subunit J	Cytoplasm	Translation regulator
1.5	7.5E-02	Q8VEM8	SLC25A3	Solute carrier family 25 member 3	Cytoplasm	Transporter
1.3	3.3E-02	P60710	ACTB	Actin beta	Cytoplasm	Other
NF-V	7.9E-04	P62259	YWHAE	Tyrosine 3-monooxygenase/tryptophan 5-monooxygenase activation protein epsilon (14-3-3 epsilon)	Cytoplasm	Other
NF-V	1.0E-02	P63044	VAMP2	Vesicle associated membrane protein 2	Plasma Membrane	Other
NF-V	1.6E-02	P46660	INA	Internexin neuronal intermediate filament protein alpha	Cytoplasm	Other
NF-V	3.7E-02	Q9QYM9	TMEFF2	Transmembrane protein with EGF like and two follistatin like domains 2	Cytoplasm	Other
NF-V	4.1E-02	Q6PHN9	RAB35	RAB35, member RAS oncogene family	Cytoplasm	Enzyme
NF-V	6.0E-02	P19246	NEFH	Neurofilament heavy	Cytoplasm	Other
NF-V	6.3E-02	Q9CZ13	UQCRC1	Ubiquinol-cytochrome c reductase core protein 1	Cytoplasm	Enzyme
NF-V	1.2E-06	Q9CQQ7	ATP5PB	ATP synthase peripheral stalk-membrane subunit b	Cytoplasm	Transporter
NF-V	2.8E-06	P80317	CCT6A	Chaperonin containing TCP1 subunit 6A	Cytoplasm	Other
NF-V	2.8E-06	Q9CWS0	DDAH1	Dimethylarginine dimethylaminohydrolase 1	Cytoplasm	Enzyme

*Ratio DZP/V is fold change in DZP animals’ peptide spectral counts (SC) relative to control vehicle treated animals. NF-V, not found in vehicle samples. N = 3 animals per treatment condition, t-test. Significant results (p < 0.05) and near significant results (p < 0.1) are shown.*

### Statistics

Relevant statistical test information is described in the figure legends or within the individual methods sections. *p*-values are reported in the results section if significance is between 0.01 and <0.05 or if the data is approaching significance.

## Results

### DZP Exposure Modifies γ2-GABA_A_R and Gephyrin Levels

**FIGURE 1:**
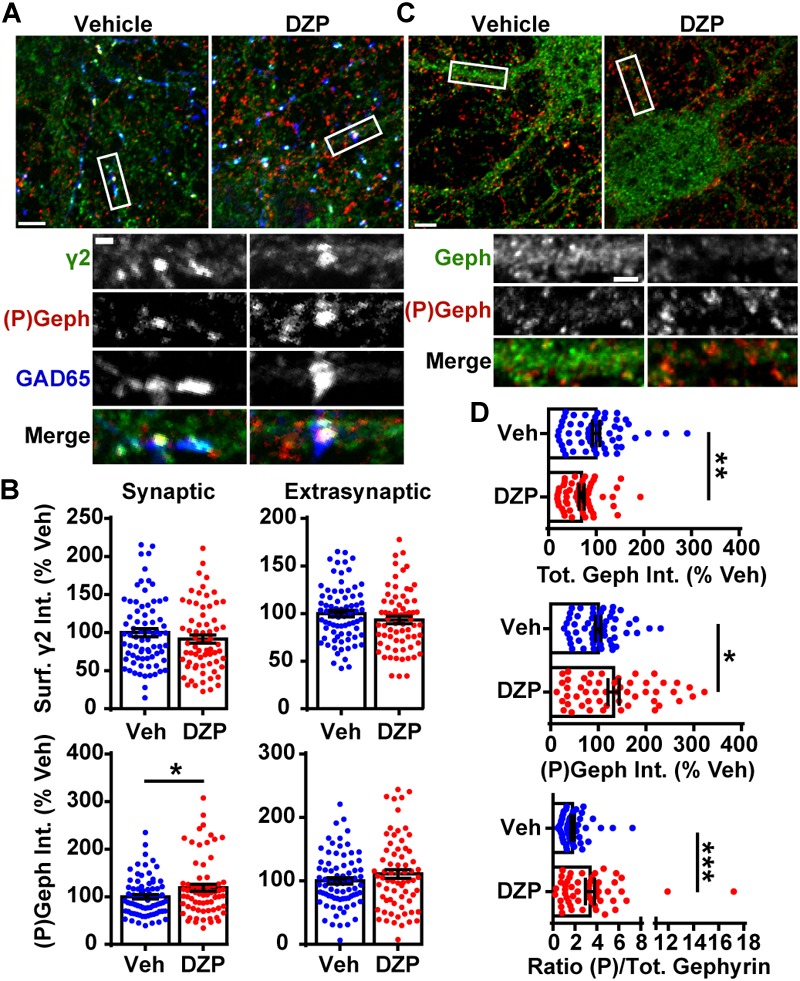
DZP downregulates gephyrin independent of γ2 surface levels. **(A)** Cortical neurons were treated for 24 h with vehicle or 1 μM DZP, then immunostained for surface γ2 GABA_A_R (green), followed by permeabilization and immunostaining for (P)Ser270 gephyrin (red), and GAD65 (blue). Panels below show enlargements of GABA_A_R synapses on dendrites. **(B)** Dendrite surface synaptic and extrasynaptic γ2 levels are not significantly altered by DZP. Synaptic phospho-gephyrin was enhanced in response to DZP (*n* = 69–74 neurons; 4 independent cultures). **(C)** Neurons were treated as in **(A)** followed by antibody staining for total gephyrin (green) and (P)Ser270 gephyrin (red). Panels below show enlargements of dendrite region. **(D)** The dendritic pool of gephyrin was decreased, while (P)Ser270 gephyrin levels were augmented, resulting in a dramatic increase in the ratio of phosphorylated gephyrin to total gephyrin (*n* = 52–59 neurons; 3 independent cultures). Int., fluorescence intensity. Image scale bars: main panels = 5 μm, enlargements = 1 μm. ^∗^*p* ≤ 0.05, ^∗∗^*p* < 0.01, ^∗∗∗^*p* < 0.001, Student’s *t*-test; error bars ± s.e.m.

**FIGURE 2:**
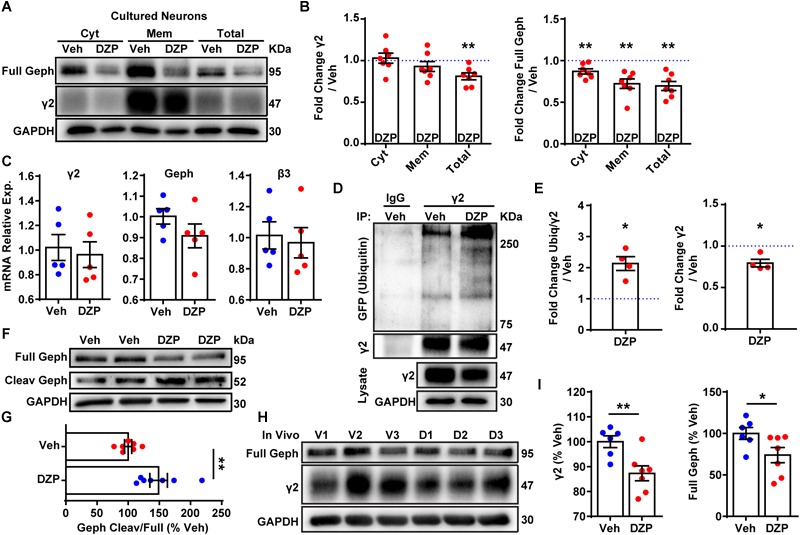
DZP induces degradation of γ2 and gephyrin *in vitro* and *in vivo*. **(A)** Cortical neurons exposed to 1 μM DZP or vehicle for 24 h were subjected to membrane fraction and western blot analysis. Full Geph = full-length gephyrin. **(B)** Total γ2 subunit and cytosolic, membrane, and total gephyrin were significantly reduced after DZP (*n* = 7 independent cultures). **(C)** Quantitative RT-PCR revealed no change in γ2 subunit, β3 subunit or gephyrin mRNA expression following 24 h DZP *in vitro* (*n* = 5 independent cultures). **(D)** GFP-ubiquitin transfected neurons were treated with vehicle or DZP for 12 h. Lysates were immunoprecipitated with control IgG or γ2 antibody, followed by blotting with anti-GFP, γ2 and GAPDH. **(E)** DZP treatment increased the levels of γ2 ubiquitin conjugates and decreased γ2 total levels. **(F,G)** DZP treatment enhanced the ratio of cleaved gephyrin fragments/full length gephyrin (*n* = 7 biological replicates/dishes from 4 independent cultures). Full, full-length gephyrin; Cleav, cleaved gephyrin. **(H)** Western blots of cortical tissue collected from mice 12 h after a single IP injection of 10 mg/kg DZP or vehicle. **(I)** γ2 subunit and full-length gephyrin levels are significantly reduced in DZP-treated animals (6–7 mice per condition). [^∗^*p* ≤ 0.05, ^∗∗^*p* < 0.01, paired *t*-test **(B,E)**, Student’s *t*-test **(C,G,I)**; error bars ± s.e.m].

We first examined if DZP exposure reduced surface levels of γ2-GABA_A_Rs and altered gephyrin Ser270 phosphorylation in cortical neurons by immunofluorescence (Figure 1A). Cortical neurons were treated for 24 h with vehicle or 1 μM DZP, then immunostained for surface γ2, followed by permeabilization and immunostaining with GAD65 (glutamic acid decarboxylase 65, a marker for presynaptic GABAergic terminals) and the phospho-Ser270 specific gephyrin mAb7a antibody (Kuhse et al., 2012; Kalbouneh et al., 2014). Image analysis identified no sizable change in surface synaptic (91.6 ± 5.3%) or extrasynaptic (93.3 ± 3.8%) γ2 intensity in DZP treated neurons relative to control, but DZP induced a significant 18.9 ± 7.4% (*p* = 0.033) increase in synaptic phospho-gephyrin (Figure 1B). No change in extrasynaptic phosphorylated Ser270 gephyrin was measured. We repeated this DZP treatment and examined total and phospho-gephyrin levels in dendrites (Figure 1C). Again DZP significantly enhanced phospho-Ser270 gephyrin compared to vehicle (132 ± 12%; *p* = 0.013), while a decrease in overall gephyrin levels was found (69.7 ± 5.4%) (Figure 1D). Accordingly, the mean ratio of phospho/total gephyrin was 78.1 ± 21% higher following DZP (Figure 1D). Complimentary biochemical studies using membrane fractionation were used to compare cytosolic, membrane, and total protein pools in cortical neurons. In agreement with immunofluorescence data, membrane levels of γ2 (0.929 ± 0.06) were not reduced after 1 μM DZP, although the total pool of γ2 was diminished (0.793 ± 0.07) (Figure 2A,B) compared to vehicle. Cytosolic levels of γ2 (1.03 ± 0.06) were also unchanged. Comparatively, DZP reduced full-length gephyrin in every compartment measured relative to control (cytosol: 0.871 ± 0.03; membrane: 0.722 ± 0.06, total: 0.695 ± 0.05). We confirmed the integrity of our fractions using cytosolic and membrane specific markers (Supplementary Figure S2).

Next we assessed if the decrease in gephyrin and γ2 total levels at 24 h was a result of altered gene expression. qRT-PCR experiments revealed no difference in gephyrin (*p* = 0.206), γ2, or control GABA_A_R β3 subunit mRNA levels between vehicle and DZP treated neurons (Figure 2C). To determine if post-translational modification of γ2 also occurs coincident with decreased γ2 protein levels, we examined ubiquitination of γ2 in response to DZP exposure. We reasoned that changes in ubiquitination of γ2 would likely precede the loss of total γ2 seen at 24 h (Figure 2A,B). GFP-ubiquitin transfected cortical neurons were treated with vehicle or 1 μM DZP for 12 h. Neurons were lysed under denaturing conditions to isolate the γ2 subunit from the receptor complex (Supplementary Figure S3). Immunoprecipitation of the γ2 subunit revealed a 2.13-fold increase (*p* = 0.015) in ubiquitination in DZP treated neurons relative to vehicle (Figure 2D,E). Furthermore, just as observed with 24 h DZP treatment, a reduced total pool of γ2 was also found at 12 h (*p* = 0.020) (Figure 2D,E). Notably, this is the first demonstration of endogenous γ2 ubiquitination occurring in neurons (previous findings were of recombinant receptors in HEK cells) (Arancibia-Carcamo et al., 2009; Jin et al., 2014). To investigate mechanisms underlying reduced full-length gephyrin levels, we examined gephyrin cleavage. Gephyrin is degraded post-translationally by the protease calpain-1 (Tyagarajan et al., 2013; Costa et al., 2015; Kumar et al., 2017), and gephyrin Ser270 phosphorylation promotes cleavage by calpain-1 (Tyagarajan et al., 2013). Consistent with the enhanced gephyrin Ser270 phosphorylation (Figure 1) and reduced full-length levels (Figure 1, 2) we found a significant increase in the ratio of cleaved/full length gephyrin after 24 h DZP *in vitro* (Figure 2F,G). We confirmed the identity of the gephyrin cleavage product using a well-characterized glutamate stimulation protocol that induces gephyrin cleavage in cultured neurons (Costa et al., 2015; Kumar et al., 2017), a process blocked by calpain-1 inhibition (Supplementary Figure S4).

Finally, we wanted to determine if similar mechanisms occur *in vivo* following DZP treatment. Prior publications show that BZDs and metabolites are not present 24 h post-injection due to rapid drug metabolism in rodents (Yoong et al., 1986; Xie and Tietz, 1992; Van Sickle et al., 2004; Markowitz et al., 2010). Furthermore, BZD uncoupling does not persist 24 h after a single dose (15 mg/kg) or 2 weeks daily DZP treatment, whereas uncoupling can be seen 12 h after a single injection, indicating this is the appropriate time point for measuring *in vivo* loss of γ2-GABA_A_R function (Holt et al., 1999). Accordingly, mice were given a single intraperitoneal (IP) injection of 10 mg/kg DZP or vehicle control, and cortex tissues were harvested 12 h later. We found DZP significantly reduced the total pool of γ2 (87.3 ± 3.0%) and full-length gephyrin (73.9 ± 9.1%; *p* = 0.046) relative to vehicle treated mice at 12 h post injection (Figure 2H,I). These findings indicate both BZD-sensitive GABA_A_Rs and full-length gephyrin are downregulated by post-translational mechanisms after initial DZP treatment *in vitro* and *in vivo* to temper potentiation of GABA_A_R function.

### DZP Enhances Intracellular Accumulation and Lysosomal Targeting of γ2-GABA_A_Rs

**FIGURE 3:**
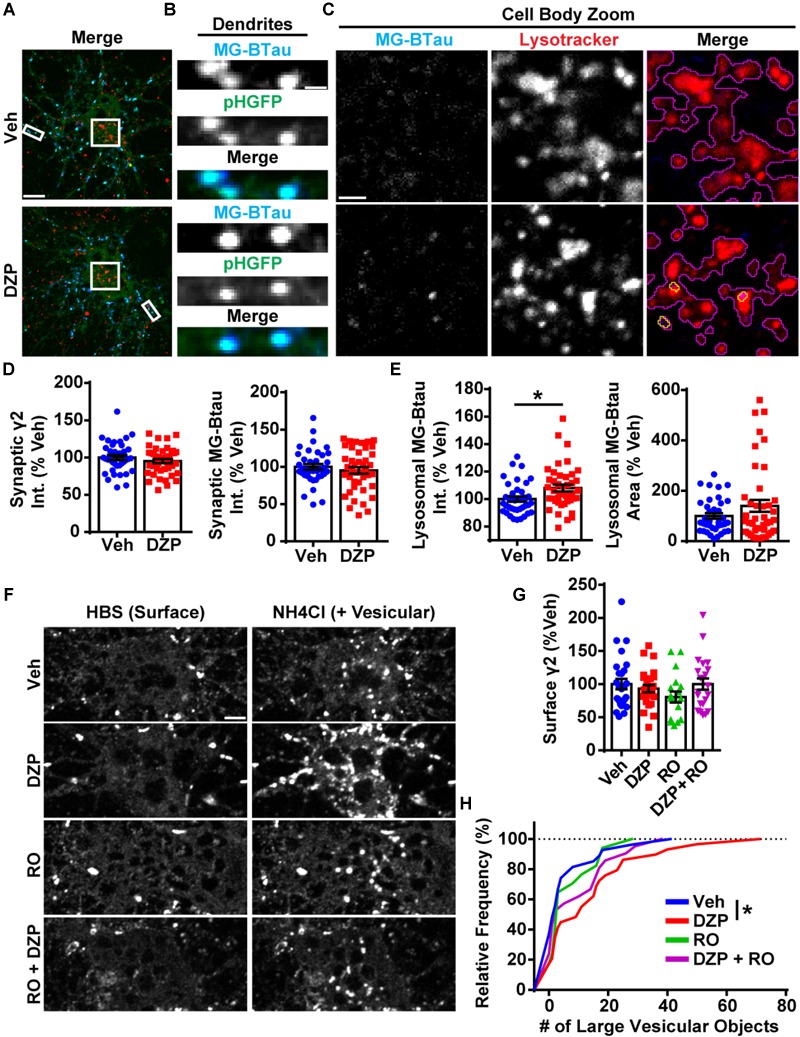
Lysosomal targeting and vesicular accumulation of γ2-GABA_A_Rs in response to DZP. **(A)** γ2^pH^FAP neurons were pretreated for 12–18 h with 1 μM DZP, then pulse-labeled with 100 nM MG-BTau dye for 2 min, and returned to conditioned media at 37°C ± DZP for 1 h. 50 nM Lysotracker dye was added at the 30 min mark to identify lysosomes. MG-BTau = blue; pHGFP = green; Lysotracker = red (*n* = 37–42 neurons; 5 independent cultures). **(B)** Dendrite zoom images show MG-BTau labeling at γ2^pH^FAP synapses. **(C)** Cell body zoom images highlighting colocalization of MG-BTau labeled GABA_A_Rs (yellow trace) at lysosomes (purple trace). **(D)** Dendrite pHGFP and MG-BTau measurements reveal surface synaptic γ2-GABA_A_R levels are not altered by DZP. **(E)** The pool of internalized MG-BTau GABA_A_Rs colocalized at lysosomes was enhanced in DZP treated neurons as measured by intensity (^∗^*p* ≤ 0.05, Student’s *t*-test; error bars ± s.e.m). **(F)** Neurons treated 20–28 h with vehicle or DZP. The DZP site antagonist Ro 15–1788 (5 μM) was added 1–2 h prior to imaging to inhibit DZP binding at GABA_A_Rs. Neurons were first imaged in HBS, and then perfused with NH_4_Cl (pH 7.4) to reveal intracellular γ2^pH^FAP receptors. DZP treated neurons accumulated more γ2-GABA_A_Rs in large vesicular structures compared to vehicle (*n* = 20–27 neurons; 3–4 independent cultures). **(G)** Surface intensity of γ2^pH^FAP was not different between treatments (one-way ANOVA; error bars ± s.e.m). **(H)** DZP-treated neurons more frequently demonstrated accumulation of γ2^pH^FAP in large vesicles (^∗^*p* ≤ 0.05 Kolmogorov–Smirnov statistical test). Int., fluorescence intensity. Scale bars in μm: **(A)** = 10; **(B)** = 1; **(C)** = 2, **(F)** = 5.

We then investigated if surface γ2-containing GABA_A_Rs are more frequently targeted to lysosomes after DZP exposure by live-imaging. For these experiments we used our recently characterized optical sensor for synaptic GABA_A_R (γ2^pH^FAP). This dual reporter is composed of a γ2 subunit tagged with an N terminal pH-sensitive GFP, myc, and the fluorogen-activating peptide DL5 (Lorenz-Guertin et al., 2017). The pH-sensitive GFP tag selectively identifies cell surface GABA_A_Rs and the DL5 FAP binds malachite green (MG) dye derivatives including MG-BTau (Szent-Gyorgyi et al., 2008, 2013; Pratt et al., 2017). MG-BTau is cell impermeable and non-fluorescent until bound by DL5. Upon binding, MG-BTau fluoresces in the far red spectral region (∼670 nM). This FAP-dye system allows for selective labeling of surface γ2-containing GABA_A_Rs which can then be tracked through various phases of trafficking (Lorenz-Guertin et al., 2017). As previously shown, γ2^pH^FAP GABA_A_Rs are expressed on the neuronal surface, form synaptic clusters, do not perturb neuronal development and show equivalent functional responsiveness to GABA and DZP both in the absence and presence of MG dyes (Lorenz-Guertin et al., 2017). We transfected neurons with γ2^pH^FAP and treated them with DZP for 8–16 h. Neurons were then pulse-labeled with 100 nM MG-BTau dye and returned to conditioned media at 37°C ± DZP for 1 h. The lysosomal inhibitor leupeptin (200 μM) and the lysosomal specific dye, Lysotracker (50 nM), were added after 30 min. At the end of the incubation, neurons were washed in 4°C saline to inhibit trafficking and immediately used for live-imaging experiments. Representative images demonstrate MG-BTau labeled γ2^pH^FAP-GABA_A_Rs localized on the cell surface (Figure 3A) and at synaptic clusters on dendrites (Figure 3B) based on colocalization with surface specific pHGFP signal. MG-BTau further reveals internalized receptors at lysosomes (Figure 3C). Image quantification showed synaptic γ2-GABA_A_R intensity remained largely unchanged (Figure 3D). Importantly, we found a significant 8.0 ± 2.5% (*p* = 0.015) enhancement in the mean intensity of GABA_A_Rs labeled with MG-BTau at lysosomes following DZP (Figure 3E). The area of GABA_A_Rs colocalized at lysosomes trended toward an increase in DZP treated cells (140.2 ± 23.6%; *p* = 0.144) but did not reach significance.

We complemented these lysosomal targeting studies with an NH_4_Cl live-imaging approach that allows us to compare the ratio of cell surface vs. intracellular GABA_A_Rs in living neurons. γ2^pH^FAP expressing neurons were treated with vehicle or DZP for 24 h. Additional control groups included the BZD antagonist Ro 15-1788 (1–2 h) to reverse the effects of DZP. Neurons were actively perfused with HEPES buffered saline (HBS) treatment and an initial image was taken of surface pHGFP receptor signal (Figure 3F). Neurons were then exposed to pH 7.4 NH_4_Cl solution to neutralize the pH gradient of all intracellular membrane compartments, revealing internal pools of γ2 containing GABA_A_Rs. Analysis revealed no change in surface γ2 levels between treatments (Figure 3G) consistent with Figure 1, 2. However, the number of large intracellular vesicles (circular area ∼0.75 μm) containing receptors was significantly enhanced (*p* = 0.047) (Figure 3H), consistent with increased localization in intracellular vesicles. Ro 15-1788 and DZP + Ro 15-1788 treated neurons were not significantly different from vehicle. Overall, these findings suggest γ2-GABA_A_R ubiquitination, intracellular accumulation, lysosomal targeting and degradation are part of the adaptive response to DZP.

### Surface Levels of Synaptic α2/γ2 GABA_A_R Are Decreased Following DZP

**FIGURE 4:**
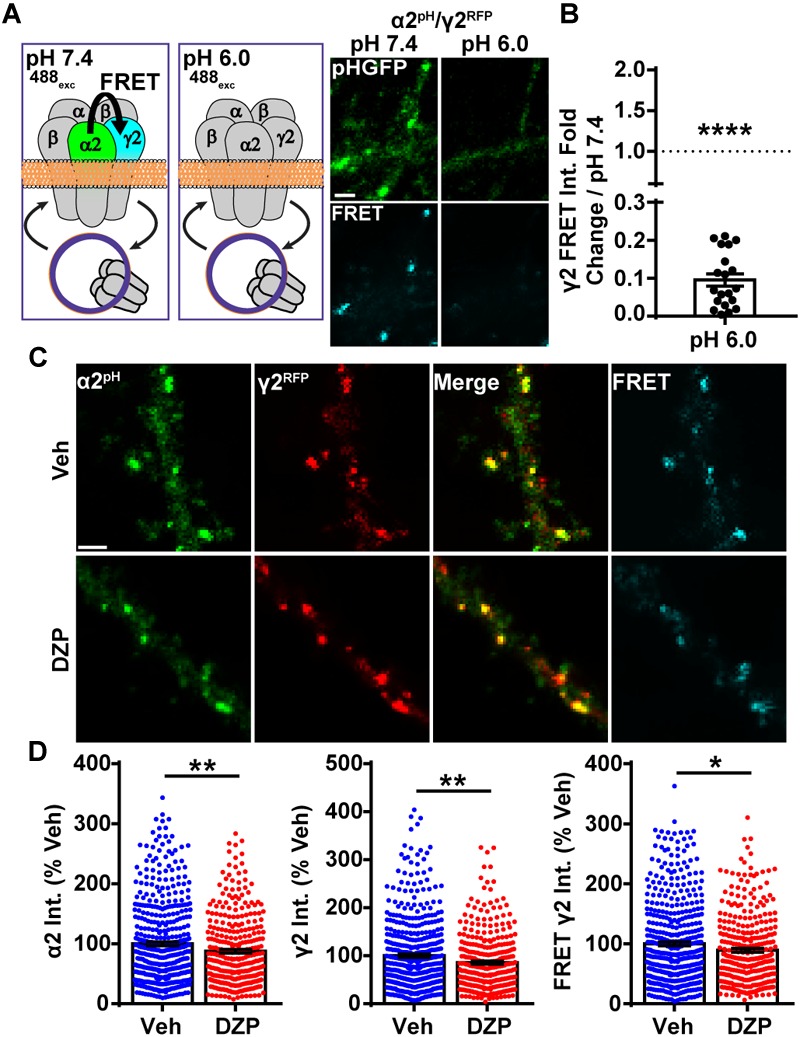
Intermolecular FRET reveals decreased dendritic synaptic α2/γ2 surface GABA_A_Rs after DZP. **(A)** Diagram and time-series images of cortical neurons expressing donor α2^pH^ (green) and acceptor γ2^RFP^ during imaging at pH 7.4 and pH 6.0. Surface α2^pH^ (green) signal and intermolecular FRET (teal) between α2/γ2 subunits occurs at pH 7.4, but is eliminated by brief wash with pH 6.0 extracellular saline and quenching of the α2^pH^ donor pHGFP fluorescence. **(B)** Quantification of relative FRET at pH 7.4 and pH 6.0 (*n* = 20 synapses). **(C)** Neurons α2^pH^ (green) and γ2^RFP^ (red) were treated with vehicle or DZP for 20–28 h ± and then subjected to live-imaging. For each cell, an initial image used 488 nm laser excitation to identify surface α2^pH^ and FRET γ2^RFP^. A second image was taken immediately afterwards to acquire γ2^RFP^ total levels (561 nm laser excitation). Dendritic lengths show multiple synaptic clusters with α2/γ2 surface GABA_A_Rs. **(D)** Synaptic cluster intensity quantification of α2^pH^, γ2^RFP^, and FRET γ2^RFP^ (at least 15 synapses per cell; *n* = 335–483 synapses; 6 independent cultures). Int., fluorescence intensity. Image scale bars = 2 μm [^∗^*p* ≤ 0.05, ^∗∗^*p* < 0.01, ^∗∗∗∗^*p* < 0.0001, paired *t*-test **(B)**, Student’s *t*-test **(D)**; error bars ± s.e.m].

Despite the increase in ubiquitination and lysosomal targeting of γ2-GABA_A_Rs after DZP, we did not detect decreased overall surface or synaptically localized γ2 levels. This suggested two possibilities, one being that a slight decrease in surface γ2-GABA_A_Rs could be challenging to detect with current methods (DZP treated cells total γ2 levels 80% of control in cultured cortical neurons; 85% *in vivo*). Alternatively, there could be an increase in γ2 subunit assembly with BZD-insensitive α subunits (γ2α4β) (Wafford et al., 1996) with a concomitant reduction in surface levels of BZD-sensitive receptors (γ2α1/2/3/5β). Our previous work showed 24 h BZD exposure in hippocampal neurons causes decreased total and surface levels of the α2 GABA_A_R subunit via lysosomal mediated degradation, without any changes in receptor insertion or removal rate (Jacob et al., 2012). To determine if α2/γ2 GABA_A_Rs are specifically decreased by DZP treatment, we developed an intermolecular FRET assay, using pH-sensitive GFP tagged α2^pH^ (Tretter et al., 2008) as a donor fluorophore and a red fluorescent protein (RFP) tagged γ2 subunit (γ2^RFP^) as an acceptor. FRET is an accurate measurement of molecular proximity at distances of 10–100 Å and is highly efficient if donor and acceptor are within the Förster radius, typically 30–60 Å (3–6 nm), with the efficiency of FRET being dependent on the inverse sixth power of intermolecular separation (Förster, 1965). Synaptic GABA_A_Rs exist as five subunits assembled in γ2-α-β-α-β order forming a heteropentameric ion channel (Figure 4A). We first expressed α2^pH^ and γ2^RFP^ in neurons and examined their ability to participate in intermolecular FRET. Photobleaching of the acceptor γ2^RFP^ channel enhanced donor α2^pH^ signal (Supplementary Figure S5), confirming energy transfer from α2^pH^ to γ2^RFP^. Next, we confirmed measurable FRET only occurs between α2^pH^/γ2^RFP^ in surface GABA_A_R at synaptic sites; FRET was blocked with quenching of donor α2^pH^ when the extracellular pH was reduced from 7.4 to 6.0 (Figure 4A,B). Following FRET assay validation, α2^pH^/γ2^RFP^ GABA_A_R expressing neurons were treated for 24 h with vehicle or DZP and examined for total synaptic α2^pH^ and γ2^RFP^ fluorescence as well as the γ2 FRET signal (Figure 4C). These studies identified a DZP-induced reduction in synaptic α2 (-12.6%), synaptic γ2 (-14.3%) and diminished association of α2 with γ2 in synaptic GABA_A_Rs as measured by decreased FRET γ2 signal (-10.6%; *p* = 0.024) (Figure 4D). In summary, this sensitive FRET method indicates that cortical neurons show a similar susceptibility for α2 subunit downregulation by BZD treatment as seen in hippocampal neurons (Jacob et al., 2012). Furthermore it identifies a DZP-induced decrease in a specific pool of surface synaptic BZD-sensitive γ2-GABA_A_R.

### Selective ERK Inactivation After DZP Treatment

**FIGURE 5:**
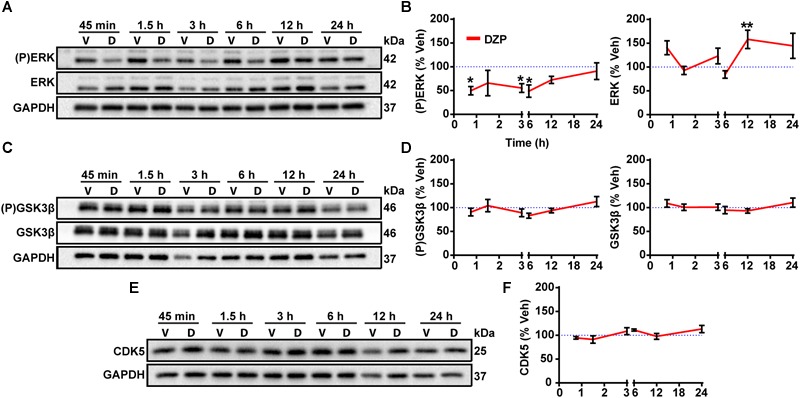
Gephyrin regulating kinases following DZP treatment. DIV 16 cortical neurons were treated with vehicle (V) or DZP (D) at multiple time points. Known kinase regulators of gephyrin synaptic clustering were tested for total protein levels and activation status with phospho-specific antibodies as indicated. (P)ERK and total ERK **(A,B)**, (P)GSK3β and total GSK3β **(C,D)** and CDK5 **(E,F)** western blot and quantification (*n* = 4 independent cultures). DZP strongly inhibited ERK phosphorylation across multiple time points while GSK3β activation and total levels did not change. CDK5 total levels were also unchanged by DZP treatment. (^∗^*p* ≤ 0.05, ^∗∗^*p* < 0.01, Two-way ANOVA followed by Sidak’s multiple comparisons test; error bars ± s.e.m).

To gain additional mechanistic insight into the molecular mechanisms controlling phosphorylation and degradation of gephyrin observed in Figure 1, 2, we performed a DZP time series experiment to measure changes in expression or activation of the gephyrin regulating kinases ERK, GSK3β, and CDK5. CDK5 and GSK3β phosphorylate gephyrin at the Ser270 site (Tyagarajan et al., 2011; Kalbouneh et al., 2014), while ERK phosphorylates a neighboring Ser268 residue (Tyagarajan et al., 2013). We first measured ERK activation by examining ERK phosphorylation across time points. DZP treatment caused a significant decrease in ERK phosphorylation at 45 min (-50.2%), 3 h (-44.5%) and 6 h (-51.2%), with a recovery in phosphorylation to vehicle levels occurring around 12 and 24 h (Figure 5A,B). Total ERK levels were unchanged after DZP, except for a significant enhancement in expression at the 12 h time point, coinciding with recovery of ERK phosphorylation. We did not detect a change in the phosphorylation or total levels of GSK3β (Figure 5C,D) or expression of CDK5 (Figure 5E,F). This data indicates that kinases involved in gephyrin phosphorylation at Ser270 do not demonstrate global changes after DZP, suggesting that the kinases may be recruited to gephyrin, or that an unknown phosphatase responsible for dephosphorylating Ser270 is inhibited after DZP exposure. Conversely, ERK inactivation by DZP is predicted to decrease phosphorylation of the functionally relevant Ser268 site of gephyrin, which has also been implicated in gephyrin synaptic remodeling (Tyagarajan et al., 2013). Gephyrin point mutant studies suggest reduced phosphorylation at Ser268 coupled with enhanced Ser270 phosphorylation, or the inverse, promotes calpain-1 degradation and scaffold remodeling (Tyagarajan et al., 2013). This data provides evidence that a known kinase pathway responsible for fine-tuning GABA_A_R synapse dynamics (Brady et al., 2017) and scaffold (Tyagarajan et al., 2013) is robustly inactivated by DZP.

### Synaptic Exchange of γ2-GABA_A_Rs and Gephyrin Are Accelerated After Prolonged DZP Treatment

**FIGURE 6:**
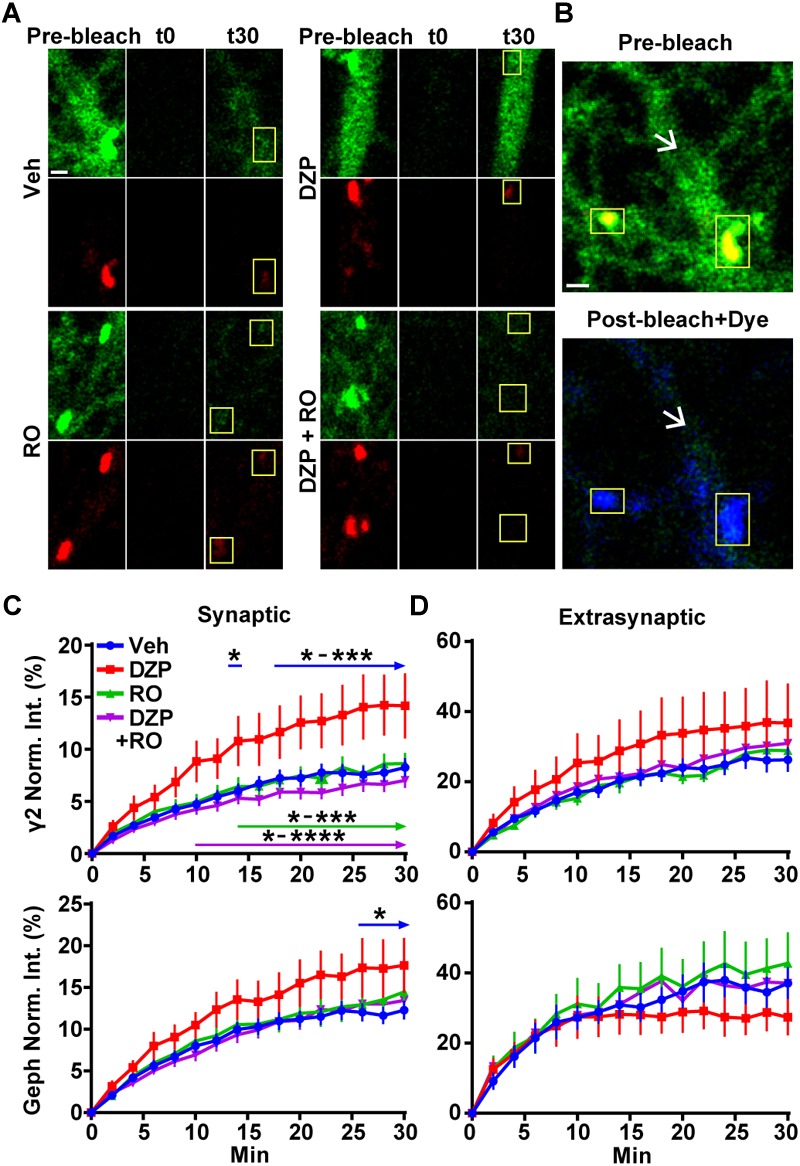
Prolonged DZP exposure accelerates γ2 GABA_A_R and gephyrin synaptic exchange. **(A)** Neurons expressing γ2^pH^FAP GABA_A_R (green) and RFP-gephyrin (red) were treated with vehicle or DZP for 20–28 h ± Ro 15-1788 for the last 1–2 h. Neurons were imaged at 37°C in constant presence of treatment. Initial image of dendrites taken prior to photobleaching (Pre-bleach), then imaged post-bleach (t0) every 2 min for 30 min. Images of dendritic regions show synaptic cluster sites (yellow boxes) and extrasynaptic regions. **(B)** 10 nM MG-BTau dye (blue) was added immediately after bleaching events in **(A)** to resolve bleached γ2^pH^FAP GABA_A_Rs and provide spatial accuracy for time series measurements. Panels show enlargement of vehicle dendritic regions identified in **(A)**. Yellow boxes indicate synaptic clusters and arrows indicate extrasynaptic region seen by pHGFP fluorescence in pre-bleach (green) followed by post-bleach labeling with Mg-Btau (blue). **(C,D)** Fluorescence recovery of γ2 GABA_A_R and gephyrin measured at synaptic sites and extrasynaptic sites from **(A)**. Synapse = γ2^pH^FAP cluster colocalized with gephyrin cluster. Int., fluorescence intensity. Image scale bars = 1 μm (^∗^*p* ≤ 0.05, ^∗∗^*p* < 0.01, ^∗∗∗^*p* < 0.001, ^∗∗∗∗^*p* < 0.0001, two-way ANOVA; Tukey’s multiple comparisons test; 4–8 synapses and one 10 μm extrasynaptic region per cell; *n* = 51–56 synapses from 16 neurons per treatment; 4 independent cultures; error bars ± s.e.m).

We previously found 24 h BZD exposure reduces the amplitude of miniature inhibitory postsynaptic currents (mIPSCs) (Jacob et al., 2012), suggesting changes in synaptic GABA_A_R function. Having identified both reductions in full-length gephyrin (Figure 1, 2) and BZD sensitive GABA_A_Rs (Figure 2, 4), we next tested if DZP treatment altered the synaptic retention properties of gephyrin and/or GABA_A_Rs. Neurons expressing γ2^pH^FAP and RFP-gephyrin were used for live-imaging fluorescence recovery after photobleaching experiments (FRAP) to measure synaptic and extrasynaptic exchange following exposure to vehicle, 1 μm DZP, 5 μm Ro 15-1788, or DZP + Ro 15-1788. After an initial image was taken, dendrites were photobleached, and signal recovery was measured every 2 min over 30 min at synaptic sites and extrasynaptic regions (Figure 6A synapses panel; Figure 6B larger dendritic region with white arrows denoting extrasynaptic region). MG-BTau dye was added directly after the photobleaching step to immediately re-identify the photobleached surface synaptic GABA_A_Rs, and improve spatial measurements (Figure 6B). These experiments revealed synaptic γ2 turnover rates were nearly doubled in DZP treated neurons, a process reversed by Ro 15-1788 co-treatment (Figure 6C). DZP also accelerated gephyrin synaptic exchange rates compared to vehicle, with Ro 15-1788 co-treatment restoring exchange to control levels. No significant correlation was found between cluster area measured and fluorescence recovery rates of γ2 and gephyrin across all conditions, suggesting synaptic exchange rate is independent of cluster size (Supplementary Figure S6). Moreover, no statistical difference was found in γ2 or gephyrin extrasynaptic exchange rates (Figure 6D). These findings suggest concurrent reduction of gephyrin and GABA_A_R synaptic confinement is a compensatory response to mitigate prolonged DZP potentiation of GABA_A_Rs.

### Co-immunoprecipitation and Quantitative Proteomics of γ2 GABA_A_R Following DZP Injection

We sought to observe DZP-induced changes in receptor trafficking *in vivo*. As an orthogonal approach, we utilized label-free quantitative proteomics to measure changes in the quantities of proteins associated with γ2-GABA_A_Rs in the cortex of mice after DZP. Cortical tissue was collected from DZP- or vehicle-treated mice 12 h post injection, lysed, and immunoprecipitated with anti-γ2 subunit antibody or IgG control. Following label-free mass spectrometry analysis, spectrum counts were used to assess relative abundance of γ2-associated proteins. A total of 395 proteins were identified using our inclusion criteria: minimum of two peptides; identified in at least three samples overall or in two of three samples in a specific treatment group; demonstrated at least 3:1 enrichment over IgG control across at least three samples overall (Supplementary Dataset 1). The relative abundance of γ2-GABA_A_R associated proteins in the DZP group compared to vehicle was used to determine which proteins were increased (Table 1) or decreased (Table 2). As a result we identified 46 proteins with elevated levels of interaction with γ2-GABA_A_Rs, including 10 proteins that were only found in the DZP treated group (Table 1, not found in vehicle samples, NF-V). Notably, we found a significant (*p* < 0.05) increase in γ2 association with 14-3-3 protein family members tyrosine 3-monooxygenase/tryptophan 5-monooxygenase activation protein gamma (also known as 14-3-3γ) and tyrosine 3-monooxygenase/tryptophan 5-monooxygenase activation protein epsilon (also known as 14-3-3𝜀), the phosphatase protein phosphatase 3 catalytic subunit alpha (also known as calcineurin/PPP3CA) and a near significant increase in the GABA_A_R α5 subunit (*p* = 0.057), suggesting DZP induced changes in GABA_A_R surface trafficking (Qian et al., 2012; Nakamura T. et al., 2016), synaptic retention (Bannai et al., 2009, 2015; Muir et al., 2010; Niwa et al., 2012; Eckel et al., 2015), and receptor composition (van Rijnsoever et al., 2004). In contrast, 23 proteins were found to co-immunoprecipitate with γ2 less in DZP animals relative to control, seven of which were only present in the vehicle treatment group (Table 2, not found in DZP, NF-DZP). Interestingly, the calcium-sensitive kinase CaMKIIα, which can regulate GABA_A_R membrane insertion, synaptic retention and drug binding properties (26, 70–72), was found to be significantly decreased in interaction with γ2-GABA_A_R following DZP injection *in vivo*.

**Table 2:** Proteins demonstrating decreased association with γ2-GABA_A_Rs after DZP *in vivo* by mass spectrometry.

Ratio DZP/V	*P*-value	UniProtKB	Gene ID	Entrez gene name	Location	Type(s)
0.2	3.4E-02	P62717	RPL18A	Ribosomal protein L18a	Cytoplasm	Other
0.2	3.9E-02	P62874	GNB1	G protein subunit beta 1	Plasma Membrane	Enzyme
0.2	4.2E-02	Q60900-2	ELAVL3	ELAV like RNA binding protein 3	Nucleus	Other
0.2	5.8E-02	Q920I9	WDR7	WD repeat domain 7	Cytoplasm	Other
0.3	2.0E-02	P53026	RPL10A	Ribosomal protein L10a	Nucleus	Other
0.3	3.4E-02	Q91VM5	Rbmxl1	RNA binding motif protein, X-linked like-1	Nucleus	Other
0.4	6.9E-02	P49312	Hnrnpa1	Heterogeneous nuclear ribonucleoprotein A1	Nucleus	Other
0.4	8.5E-02	Q8BG05	Hnrnpa3	Heterogeneous nuclear ribonucleoprotein A3	Nucleus	Transporter
0.4	7.9E-02	Q922F4	TUBB6	Tubulin beta 6 class V	Cytoplasm	Other
0.5	5.2E-02	P62334	PSMC6	Proteasome 26S subunit, ATPase 6	Nucleus	Peptidase
0.5	2.9E-04	P11798	CAMK2A	Calcium/calmodulin dependent protein kinase II alpha	Cytoplasm	Kinase
0.5	3.6E-02	E9PV14	EPB41L1	Erythrocyte membrane protein band 4.1 like 1	Plasma Membrane	Other
0.7	6.8E-02	P16330	CNP	2′,3′-cyclic nucleotide 3′ phosphodiesterase	Cytoplasm	Enzyme
0.7	7.6E-02	O35643	AP1B1	Adaptor related protein complex 1 subunit beta 1	Cytoplasm	Transporter
0.8	1.4E-02	P68369	TUBA1A	Tubulin alpha 1a	Cytoplasm	Other
0.8	8.0E-02	P52480	PKM	Pyruvate kinase M1/2	Cytoplasm	Kinase
NF-DZP	1.5E-02	P61358	RPL27	Ribosomal protein L27	Cytoplasm	Other
NF-DZP	3.5E-02	Q9Z1X4-3	ILF3	Interleukin enhancer binding factor 3	Nucleus	Transcription regulator
NF-DZP	5.5E-02	Q80UJ0	ELAVL2	ELAV like RNA binding protein 2	Cytoplasm	Other
NF-DZP	2.8E-06	Q3UHB8	CCDC177	Coiled-coil domain containing 177	Other	Other
NF-DZP	2.8E-06	P49615	CDK5	Cyclin dependent kinase 5	Nucleus	Kinase
NF-DZP	2.8E-06	Q6ZWV3	RPL10	Ribosomal protein L10	Cytoplasm	Translation regulator
NF-DZP	2.8E-06	Q9CQ69	UQCRQ	Ubiquinol-cytochrome c reductase complex III subunit VII	Cytoplasm	Enzyme

*Ratio DZP/V is fold change in DZP animals’ peptide SC relative to control vehicle treated animals. NF-DZP, not found in DZP samples. N = 3 animals per treatment condition, t-test. Significant results (p < 0.05) and near significant results (p < 0.1) are shown.*

### Bioinformatics Analysis of the γ2 GABA_A_R Interactome

**FIGURE 7:**
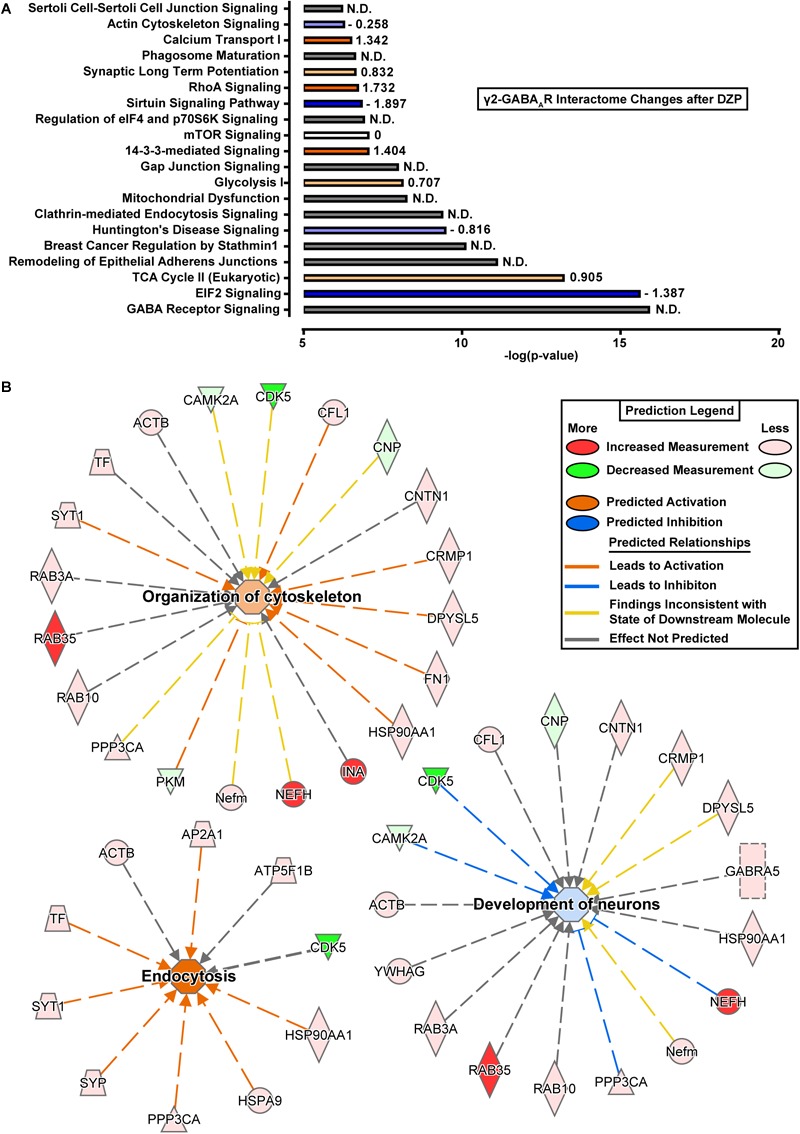
Ingenuity pathway analysis reveals shifts in protein interaction networks following DZP exposure. **(A)** Canonical pathways found to be enriched with γ2-GABA_A_Rs and differentially expressed following DZP administration *in vivo*. Enriched pathways with –log(*p*-value) greater than 6.2 were considered as calculated by Fisher’s exact test right-tailed. Values to right of bars represent pathway activation *z*-score. Positive *z*-score represents predicted upregulation of a pathway (orange), negative *z*-score predicts inhibition (blue), *z*-score = 0 represents no change in pathway (white), while not determined (N.D.) conveys the analysis program was unable to determine a significant change (gray). Intensity of color represents size of *z*-score value. **(B)** Functional network association of select pathways when using proteins which were found to be increased or decreased with a *p* < 0.1. Major functional pathways altered by DZP include endocytosis (*z*-score = 2.626), organization of cytoskeleton (*z*-score = 0.672), and development of neurons (*z*-score = –0.293). Significant protein changes (*p* < 0.05) conserved between two or more pathways include decreased γ2-GABA_A_R association with CAMKIIα and CDK5 and enhanced association with calcineurin/PPP3CA, the intracellular trafficking protein RAB35 and the cytoskeletal protein NEFH (also known as heavy neurofilament protein). Red = increased measurement, green = decreased measurement, orange = activation of pathway, blue = inhibition of pathway, yellow = findings inconsistent with state of downstream molecule, gray = effect not predicted.

To better understand the consequences of the DZP-induced shift in the γ2-GABA_A_R protein interaction network, protein fold change data was subjected to core Ingenuity Pathway Analysis (IPA). Top enriched canonical pathways with -log(*p*-value) > 6.2 are shown in Figure 7. Notably, GABA receptor signaling pathways were highly enriched, as expected, although IPA was unable to determine pathway activation status by *z*-score analysis. γ2-GABA_A_R association with proteins involved in 14-3-3 mediated signaling and RhoA signaling pathways were greatly increased after DZP (Figure 7A, orange), while interaction with proteins involved in EIF2 signaling and sirtuin signaling pathways were reduced (Figure 7A, blue) relative to vehicle.

We further examined alterations in functional network association relevant to receptor trafficking by checking the predicted activation status of select pathways when only using proteins which were found to be increased or decreased (Tables 1, 2). Figure 7B lists γ2-GABA_A_R major functional pathways found to be altered by DZP, contributing to processes such as endocytosis (*z*-score = 2.626), organization of cytoskeleton (*z*-score = 0.672), and development of neurons (*z*-score = -0.293). Significant protein changes (*p* < 0.05) conserved between two or more pathways include decreased γ2-GABA_A_R association with CAMKIIα and CDK5 and enhanced association with calcineurin/PPP3CA, the intracellular trafficking protein RAB35 and the cytoskeletal protein NEFH (also known as heavy neurofilament protein). As an additional measurement, we performed gene ontology (GO) database analysis of proteins which were found to be increased in DZP treated mice relative to vehicle control (Table 3). GO analysis identified enrichment in γ2 association with proteins involved in intracellular trafficking and cellular localization biological pathways after DZP, consistent with IPA analysis findings. Taken together, these results suggest DZP modifies intracellular and surface trafficking of γ2-GABA_A_Rs both *in vitro* and *in vivo*.

**Table 3:** GO analysis reveals enrichment of intracellular trafficking, transport, and protein localization pathways after DZP.

GO biological process	GO term ID	Fold enrichment	*P*-value
Transport	GO:0006810	4.2	9.8E-09
Establishment of localization	GO:0051234	4.0	2.5E-08
Establishment of localization in cell	GO:0051649	6.8	2.0E-07
Localization	GO:0051179	3.3	2.5E-07
Intracellular transport	GO:0046907	7.2	1.6E-06
Regulation of localization	GO:0032879	4.3	3.7E-06
Intracellular protein transport	GO:0006886	9.1	7.8E-06
Regulation of transport	GO:0051049	5.1	2.2E-05
Cellular localization	GO:0051641	4.8	4.8E-05
Protein transport	GO:0015031	6.1	2.2E-04

*Proteins found to have enhanced association with γ2-GABA_*A*_Rs during Scaffold analysis were searched in the GO Ontology database for biological process pathway enrichments. PANTHER Overrepresentation test and Fisher’s Exact test with Bonferroni correction for multiple testing was used to determine significance.*

## Discussion

This work identifies key trafficking pathways involved in GABA_A_R neuroplasticity in response to initial DZP exposure. Using a combination of biochemical and imaging techniques, we identified total γ2 subunit levels are diminished in response to 12–24 h of DZP exposure *in vitro* and *in vivo*. Concurrent with the decrease in the overall γ2 pool, we found DZP treatment enhanced ubiquitination of this subunit. Use of an innovative optical sensor for BZD sensitive GABA_A_R (γ2^pH^FAP) in combination with MG dye pulse-labeling approaches revealed DZP exposure moderately enhanced targeting of surface γ2-GABA_A_Rs to lysosomes. Live-imaging experiments with pH 7.4 NH_4_Cl revealed increased intracellular receptor pools, providing further evidence that DZP enhances γ2-GABA_A_R lysosomal accumulation, a response reversed by BZD antagonist Ro 15-1788 treatment. We used novel intersubunit FRET based live-imaging to identify that surface synaptic α2/γ2 GABA_A_Rs were specifically decreased after DZP, suggesting these receptor complexes were subjected to ubiquitination, lysosomal targeting, and degradation. In addition to DZP modulation of receptor trafficking, the postsynaptic scaffolding protein gephyrin demonstrated significant plasticity including increased Ser270 phosphorylation and production of gephyrin proteolytic fragments, concurrent with a decrease in total and membrane full-length gephyrin levels and ERK inactivation. Given the fundamental role of gephyrin in scaffolding GABA_A_Rs and regulating synaptic confinement, we used simultaneous FRAP live-imaging of receptors and scaffold in neurons to monitor inhibitory synaptic dynamics. We found ∼24 h DZP exposure accelerates both the rate of gephyrin and GABA_A_R exchange at synapses as shown by enhanced fluorescence recovery rates. Control experiments using the BZD antagonist Ro 15-1788 were able to reverse the DZP induced loss of synaptic confinement, reducing gephyrin and GABA_A_R mobility back to vehicle levels. Finally, we used label-free quantitative mass spectrometry and bioinformatics to identify key changes in γ2-GABA_A_R protein association *in vivo* suggesting alterations in trafficking at the cell surface and intracellularly. Collectively, this work defines a DZP-induced reduction of gephyrin scaffolding coupled with increased synaptic exchange of gephyrin and GABA_A_Rs. This dynamic flux of GABA_A_Rs between synapses and the extrasynaptic space was associated with enhanced γ2-GABA_A_R accumulation in intracellular vesicles and γ2-GABA_A_R subtype specific lysosomal degradation. We propose DZP treatment alters these key intracellular and surface trafficking pathways ultimately diminishing responsiveness to DZP.

Numerous classical studies have examined gene and protein expression adaptations in GABA_A_R subunits after BZD exposure with minimal agreement that a specific change occurs (Bateson, 2002; Uusi-Oukari and Korpi, 2010; Vinkers and Olivier, 2012). Here molecular mechanistic insight is provided, through direct measurements of enhanced ubiquitination of the γ2 subunit (Figure 2), lysosomal targeting (Figure 3), reduced surface synaptic α2/γ2 GABA_A_R levels (Figure 4), and reduced synaptic confinement (Figure 6) of DZP-sensitive GABA_A_Rs. Together this suggests BZD exposure primarily decreases synaptic retention of γ2 containing GABA_A_R while downregulating surface levels of the α2 subunit. Ubiquitination of the γ2 subunit by the E3 ligase Ring Finger Protein 34 (RNF 34) (Jin et al., 2014) is the only currently known mechanism targeting internalized synaptic GABA_A_Rs to lysosomes (Arancibia-Carcamo et al., 2009). Due to the requirement of the γ2 subunit in all BZD-sensitive GABA_A_Rs, it is likely that ubiquitination of the γ2 subunit is a contributing factor for increased lysosomal-mediated degradation in response to DZP. Despite a small decrease in the γ2 total protein, changes in surface levels were not significant by biochemical approaches, consistent with evidence that γ2-GABA_A_R surface levels are tightly regulated to maintain baseline inhibition and prevent excitotoxicity. For example, in heterozygous γ2 knockout mice a 50% reduction in γ2 levels appears to be compensated by increased cell surface trafficking, resulting in only an approximately 25% reduction in BZD binding sites in the cortex and a limited reduction in synaptic GABA_A_R clusters (Crestani et al., 1999; Ren et al., 2015). In contrast, homozygous γ2 knockout mice show a complete loss of behavioral drug response to BZD and over 94% of the BZD sites in the brain (GABA binding sites unchanged) and early lethality (Gunther et al., 1995). Similarly, studies have shown that prolonged GABA_A_R agonist or BZD application increases γ2 GABA_A_R internalization in cultured neurons, while surface GABA_A_R levels are variably affected (Chaumont et al., 2013; Nicholson et al., 2018). Importantly, by using high sensitivity surface GABA_A_R intersubunit FRET measurements we were able to detect a decrease in BZD sensitive α2/γ2 GABA_A_Rs (Figure 4).

The role of inhibitory scaffolding changes in responsiveness to BZD has been largely under investigated. Phosphorylation of gephyrin at Ser270 is mediated by CDK5 and GSK3β, while a partnering and functionally relevant Ser268 site is regulated by ERK (Tyagarajan et al., 2013). DZP time series experiments revealed a global decrease in ERK phosphorylation but not GSK3β, without a change in total kinase levels of ERK, GSK3β, or CDK5 over the course of the assay (except 12 h ERK) (Figure 5). A previous model by Tyagarajan et al. (2013) using gephyrin point mutants at Ser268 and Ser270 suggested that enhanced Ser270 phosphorylation coupled with decreased Ser268 phosphorylation by ERK promotes gephyrin remodeling and calpain-1 degradation. This is consistent with the ERK inactivation measured in our data and the increase in gephyrin Ser270 phosphorylation demonstrated by immunofluorescence after DZP (Figure 1), enhanced gephyrin degradation and decreased full-length gephyrin levels (Figure 1, 2). Calpain-1 mediated gephyrin cleavage can occur within 1 min in hippocampal membranes (Kawasaki et al., 1997), and cleavage products are increased following *in vitro* ischemia at 30 min and up to 48 h following ischemic events *in vivo* (Costa et al., 2015). Gephyrin cleavage may be occurring at earlier time points than the DZP 24 h mark measured here (Figure 2F,G), coinciding with ERK dephosphorylation as early as 45 min (Figure 5A,B). One limitation of our results is that measuring total and phospho levels of these kinases does not directly address changes in association or regulation of gephyrin, although it does provide an additional piece of evidence supporting gephyrin cleavage by calpain-1 and scaffold remodeling. Accordingly, our previous work found 30 min treatment with the GABA_A_R agonist muscimol in immature neurons (depolarizing) leads to ERK/BDNF signaling and decreased Ser270 phosphorylated gephyrin levels at synapses and overall (Brady et al., 2017). Thus, ERK activation status negatively correlates with the level of phosphorylation at gephyrin Ser270.

Recent work has demonstrated 12 h DZP treatment of organotypic hippocampal slices expressing eGFP-gephyrin causes enhanced gephyrin mobility at synapses and reduced gephyrin cluster size (Vlachos et al., 2013). Here we found the synaptic exchange rate of γ2 GABA_A_Rs and gephyrin to be nearly doubled at synapses in cortical neurons after ∼24 h DZP exposure (Figure 6). γ2 extrasynaptic fluorescence recovery in DZP treated neurons was variable but also trended toward an increase relative to controls (Figure 6), which could be a result of increased diffusion of receptors out of the synaptic space. This effect occurred coincident with the formation of truncated gephyrin cleavage products (Figure 2), which has previously been shown to decrease γ2 synaptic levels (Costa et al., 2015). These findings are also consistent with our previous work showing RNAi gephyrin knockdown doubles the rate of γ2-GABA_A_R turnover at synaptic sites (Jacob et al., 2005). Later quantum dot single particle tracking studies confirmed γ2 synaptic residency time is linked to gephyrin scaffolding levels (Renner et al., 2012). Importantly, GABA_A_R diffusion dynamics also reciprocally regulate gephyrin scaffolding levels (Niwa et al., 2012), suggesting gephyrin and GABA_A_Rs synaptic residency are often functionally coupled. Accordingly, γ2 subunit and gephyrin levels both decrease in responses to other stimuli including status epilepticus (Gonzalez et al., 2013) or prolonged inhibition of IP_3_ receptor-dependent signaling (Bannai et al., 2015). Additionally, chemically induced inhibitory long-term potentiation (iLTP) protocols demonstrate gephyrin accumulation occurs concurrent with the synaptic recruitment of GABA_A_Rs within 20 min (Petrini et al., 2014). Collectively, these proteins display a high degree of interdependence across different experimental paradigms of inhibitory synapse plasticity occurring over minutes to days.

Increasing receptor synaptic retention enhances synaptic currents, while enhanced receptor diffusion via decreased scaffold interactions reduces synaptic currents. For example, reduction of gephyrin binding by replacement of the α1 GABA_A_R subunit gephyrin binding domain with non-gephyrin binding homologous region of the α6 subunit results in faster receptor diffusion rates and a direct reduction in mIPSC amplitude (Mukherjee et al., 2011). Similarly, enhanced diffusion of GABA_A_Rs following estradiol treatment also reduces mIPSCs in cultured neurons and in hippocampal slices (Mukherjee et al., 2017). In contrast, brief DZP exposure (<1 h) reduces GABA_A_R synaptic mobility (Levi et al., 2015) without a change in surface levels (Gouzer et al., 2014), consistent with initial synaptic potentiation of GABA_A_R neurotransmission by DZP. Together with our current findings, this suggests post-translational modifications on GABA_A_R subunits or gephyrin that enhance receptor diffusion are a likely key step leading to functional tolerance to BZD drugs.

It is a significant technical challenge to examine dynamic alterations in receptor trafficking occurring *in vivo*. To overcome this we examined changes in γ2-GABA_A_R protein association following DZP injection in mice using quantitative proteomics and bioinformatics analysis. This work revealed shifts toward γ2-GABA_A_R association with protein pathway networks associated with endocytosis and organization of cytoskeleton (Figure 7B and Table 3), confirming similar fluctuations in membrane and intracellular trafficking occur *in vivo* and *in vitro* after DZP. We also found that shifts in association of proteins involved in the development of neurons (CAMKIIα, CDK5, NEFH, and calcineurin/PPP3CA) suggested an inhibition in this pathway after DZP (Figure 7B). When considering all protein hits between vehicle and DZP, γ2-GABA_A_R association with proteins involved in 14-3-3 mediated signaling and RhoA signaling pathways were greatly increased after DZP (Figure 7A, orange), while interaction with proteins involved in EIF2 signaling and sirtuin signaling pathways were reduced (Figure 7A, blue). 14-3-3 proteins are heavily linked in GABA_A_R intracellular to surface trafficking (Qian et al., 2012; Nakamura T. et al., 2016), and the RhoA signaling pathway is directly involved in actin cytoskeleton organization (Negishi and Katoh, 2002) and α5-GABA_A_R anchoring (Hausrat et al., 2015), providing further evidence of GABA_A_R shifts in membrane and cytosolic trafficking after DZP exposure.

Recent inhibitory synapse proteomics studies have identified a number of new protein synaptic constituents or modulators of GABA_A_R function (Butko et al., 2013; Kang et al., 2014; Nakamura Y. et al., 2016; Uezu et al., 2016; Ge et al., 2018). We show here that proteins known to have roles in synaptic function and trafficking of membrane receptors show changes in their association with γ2-receptors. For example, the calcium-sensitive kinase CaMKIIα was found to be significantly decreased in interaction with γ2-GABA_A_R following DZP, which can regulate GABA_A_R membrane insertion, synaptic retention and drug binding properties (Churn et al., 2002; Marsden et al., 2010; Saliba et al., 2012; Petrini et al., 2014) (Table 2). Calcineurin/PPP3CA has been recognized as a key regulator of GABA_A_R synaptic retention and plasticity (Bannai et al., 2009; Muir et al., 2010; Niwa et al., 2012; Bannai et al., 2015; Eckel et al., 2015) and has been linked to the response to DZP *in vitro* (Nicholson et al., 2018). Here we provide the first evidence that DZP exposure enhances the association of calcineurin with γ2-GABA_A_Rs *in vivo*. Furthermore, DZP was found to enhance γ2 association with 14-3-3 protein family members (Table 1), which are known mediators of GABA_A_R surface and intracellular trafficking (Qian et al., 2012; Nakamura T. et al., 2016). γ2 coassembly with the GABA_A_R α5 subunit was also elevated post DZP exposure (Table 1). Interestingly, the α5 subunit is required for the development of BZD sedative tolerance in mice (van Rijnsoever et al., 2004). It is notable that our proteomic studies are in part limited by the specificity of our antibody used and general downstream effects of reduced neuronal activity. Future follow up studies using the DZP site antagonist R015-1788 will be needed to dissect the individual roles of proteins found to be significantly altered in their association with GABA_A_R, and their physiological and pharmacological importance to BZD tolerance and inhibitory neurotransmission.

Through application of novel and highly sensitive fluorescence imaging approaches combined with *in vivo* proteomics, we provide unprecedented resolution of GABA_A_R synapse plasticity induced by BZDs at both the level of the single neuron and cortex. Our study reveals that sustained initial DZP treatment diminishes synaptic BZD sensitive GABA_A_R availability through multiple fundamental cellular mechanisms: through reduction of the post-synaptic scaffolding protein gephyrin; shifts toward intracellular trafficking pathways and targeting of receptors for lysosomal degradation; and enhanced synaptic exchange of both gephyrin and GABA_A_Rs. Proteomic and bioinformatics studies using DZP-treated mouse brain tissue provide further evidence that altered γ2-GABA_A_R surface and intracellular trafficking mechanisms play a critical role to the response to DZP *in vivo*. These results define key events leading to BZD irresponsiveness in initial sustained drug exposure. Future studies utilizing this dual approach will address the neuroadaptations produced by long term BZD use to systematically identify the effects of a critical drug class that has seen a tripling in prescription numbers over the last two decades (Bachhuber et al., 2016).

## Author Contributions

JL-G and TJ designed the research. JL-G, TJ, and SW wrote and revised the manuscript. JL-G performed the biochemistry, immunoprecipitation, bioinformatics analysis and fixed and live imaging acquisition and analysis in Figure 1–6. MB performed the FRET imaging and analysis. JL-G and SD performed the tissue collection. TJ, JL-G, and SW designed the Mass spectrometry experiments. SW and the Weintraub lab performed mass spectrometry and data was analyzed by JL-G, TJ, and SW.

## Conflict of Interest Statement

The authors declare that the research was conducted in the absence of any commercial or financial relationships that could be construed as a potential conflict of interest.
